# Addressing Ethnicity in the Design and Evaluation of an Educational Intervention on Interindividual Variation in Pharmacokinetics

**DOI:** 10.1002/prp2.70073

**Published:** 2025-02-06

**Authors:** Jennifer A. Koenig, Olusola Olafuyi, Rakesh Patel

**Affiliations:** ^1^ School of Medicine University of Nottingham Nottingham UK; ^2^ School of Life Sciences University of Nottingham Nottingham UK; ^3^ Institute of Health Sciences Education, Barts and The London Faculty of Medicine and Dentistry Queen Mary University of London London UK

**Keywords:** drug metabolism, ethnicity, genetic determinism, pharmacokinetics, racial group

## Abstract

Interindividual variation in pharmacokinetics can occur due to diet, environmental or lifestyle factors, underlying pathology, and gene variants, typically single nucleotide polymorphisms (SNPs). Genetic mechanisms have received the most attention in research and education about ethnic differences in pharmacokinetics. Making this connection between genetics and ethnicity is problematic because it could reinforce the erroneous idea that there is a biological basis to ethnicity. The aim of this work was to design an educational intervention about interindividual variation in pharmacokinetics, explore how students perceive ethnicity and genetic differences prior to the educational intervention, and then assess the impact of the intervention and whether it could influence any misconceptions students might have about ethnicity and genetic similarity. Through the use of questionnaires and focus groups, we found that students typically refer to ethnicity to mean culture and place of origin, whereas in the pharmacological literature, ethnicity is synonymous with racial groups, that is, Black, White, and Asian. Prior to the educational intervention, students tended to expect a genetic mechanism for ethnic differences in drug metabolism and this was reduced after the intervention when a range of other nongenetic mechanisms were presented for interindividual variation. However, students' views about possible underlying mechanisms for ethnic differences in hypertension and about ethnicity more generally were unaffected by the intervention. This highlights the importance of reevaluating the way ethnicity is presented across the medical and medical sciences curriculums to be clear that ethnicity is socially constructed and avoid implying a biological basis.

## Graphical abstract

Students show confusion about ethnicity and genetic similarity and tend to suggest genetic mechanisms for proposed ethnic differences. Teaching reduces this but has no effect on ethnicity in other medical contexts. This has important implications for the wider curriculum to avoid inadvertently promoting a biological basis for ethnicity when it is socially constructed.

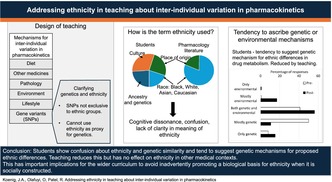

## 1 Introduction

**FIGURE 1:**
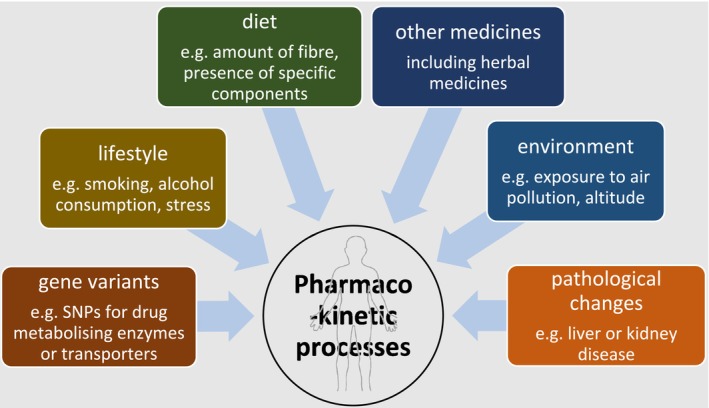
Factors that can result in interindividual variation in pharmacokinetics processes.

There are several aspects of pharmacology where ethnicity and race become relevant, for example, NICE guidelines for hypertension which recommend different classes of drugs for certain ethnic groups [1] and suggestions for dose adjustment due to pharmacokinetic differences between ethnic groups [2]. In recent years, some physiological and diagnostic measurements such as those for kidney [3] and lung function [4] have been revised after reevaluation of the evidence to remove terms that treat certain ethnic groups differently. A scoping review investigating the relationship between ethnicity and pharmacokinetic processes [5] confirmed that there is more evidence for similarities rather than differences between ethnic groups. Where there are ethnic differences, a wide range of potential underlying mechanisms have been proposed (Figure 1), with single nucleotide polymorphism (SNP) prevalence in metabolizing enzymes and drug transporter proteins identified as the most common reason [6, 7, 8]. Most pharmacology educators teach about variation in SNP prevalence rather than other factors such as diet and environment, when discussing ethnic differences in pharmacokinetics [5].

These findings from the scoping review raise several questions about genetics and ethnicity, and in particular, the context in which both are taught to students as well as any preconceived ideas held by the latter when entering higher education. Teaching about genetic mechanisms in relation to ethnicity in medical education is challenging because of the risk of inadvertently inferring a biological basis to ethnicity and race when of course these should be presented as social constructs, that is, phenomena or conventions cultivated by society [9, 10, 11, 12, 13]. Studies have shown that both high school and undergraduate students have significant misunderstandings regarding genetics: Many think, erroneously, that there is more genetic variation *between* ethnic groups *than within* ethnic groups [14, 15]. In fact, human genetic variation is continuous and does not map to racial categories [16, 17]. It is important, therefore, that any teaching about genetic mechanisms in pharmacokinetics makes clear that ethnicity is a social construct and does not have a biological basis.

This study outlines the design of an educational intervention about interindividual variation in pharmacokinetics which aims to clarify concepts of human genetic variation in relation to ethnicity and highlight the many potential underlying mechanisms for interindividual variation in pharmacokinetics. We also carried out an exploratory study using questionnaires and focus groups to probe students' understanding of genetics and perceptions of ethnicity to find out what prior knowledge and understanding students bring to the classroom and investigated the impact of our educational intervention.

## 2 Methods

### 2.1 Context and Population

Students from three undergraduate (Graduate Entry Medicine, and BSc courses in Medical Physiology and Therapeutics and Pharmacology) and one postgraduate course (MSc Drug Discovery) in a large research‐intensive University in central England were invited to participate in the study. Students across these courses were ethnically diverse with 33% identifying as Black and minority ethnic (BAME) in 2021–2022 [18].

### 2.2 Study Design

This study involved three phases: First, the design of an educational intervention on the subject of interindividual variability in pharmacokinetics; second, a pretest–posttest questionnaire design to investigate students' understanding of ethnicity and genetics separately and in the context of pharmacological applications; and third, focus groups to explore questionnaire responses in more detail.

**FIGURE 2:**
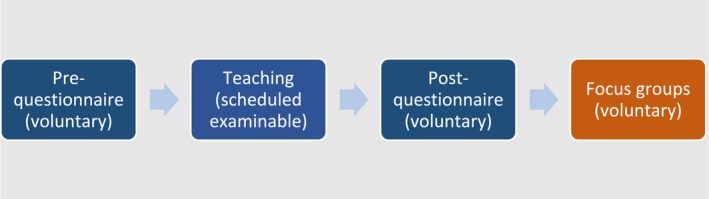
Study design.

The pretest–posttest design is summarized in Figure 2. The prequestionnaire, teaching, and postquestionnaire were carried out together in a scheduled teaching time while the focus groups were carried out between 1 and 4 weeks later.

### 2.3 Educational Intervention

An evidence‐based teaching presentation was designed based on best practices identified from a scoping review [5]. Table 1 summarizes the evidence base for each of the points made, and the teaching presentation outline is given in Supporting Information S1 “Inter‐individual variability in PK teaching resource.pdf.” Factors such as diet, smoking, alcohol, age, sex, pregnancy, presence of drug‐metabolizing enzyme or transporter variants, and isoforms that influence interindividual variability across pharmacokinetic processes were highlighted in the presentation.

**TABLE 1:** Evidence base for the teaching presentation.

Drug/drug class	Mechanism	Pharmacokinetic process	Reference
Paracetamol	Vegetarian diet	Absorption	[19]
Clopidogrel	Cytochrome P560 CYP2C19 polymorphisms	Simulation modeling of changes in metabolism by CYP enzymes	[20]
Rosuvastatin	Organic anion transporter protein OATP1b1 *1A polymorphisms	Measured overall exposure, hence multiple processes may be affected including clearance and bioavailability	[21]
Warfarin	Suggested genetic mechanism but did not measure allele frequency	None specified, dose requirement was measured, and varies with ethnicity	[22]
Several	Alcohol consumption	Absorption, metabolism	[23]
Several	Pregnancy	Absorption, distribution, metabolism, excretion	[24, 25]

When discussing ethnicity, key points were emphasized to mitigate the chances of any misconceptions about the topic after the event:

- Individuals vary due to a variety of factors both genetic and nongenetic.
- We did not foreground one mechanism over any other.
- Allele prevalence is not exclusive to a particular ethnic group, for example, the organic anion transporter OATP1B1 variants [26, 27], and therefore ethnicity should not be used as a proxy for genetics
- There is as much genetic diversity within ethnic groups as between them.

### 2.4 Questionnaire

A prequestionnaire was prepared by adapting a questionnaire by Jamieson and Radick [28]; see Supporting Information S2 for the questionnaire. Participants were invited to complete the questionnaire immediately prior to a mandatory tutorial about interindividual variation in pharmacokinetics that was arranged as part of all four courses above.

Participants were reminded that attending the teaching was not contingent on completing the questionnaire. A postquestionnaire was administered immediately after the educational intervention, with participants again being reminded about the voluntary nature of the questionnaire.

The prequestionnaire included three sections:

1. “Background information” asked about prior studies of biology and genetics.
2. “Your knowledge” contained a short true–false genetics knowledge quiz adapted from Jamieson and Radick [28].
3. “Your opinions” included questions asking students how they understand the term ethnicity, and statements about genes, disease, inheritance, and ethnicity, adapted from Jamieson and Radick [28], with 5‐point Likert‐scale responses indicating level of agreement. Finally, there were two additional questions about the genetic and/or environmental aspects of drug action.

Sections 1 and 2 of the prequestionnaire were included to provide a richer understanding of our students and their background and prior knowledge. The postquestionnaire included Section 3 only. Questionnaires were delivered through JISC Online Surveys (www.onlinesurveys.ac.uk) and responses were anonymous. Responses to the pre‐ and postquestionnaires were matched for subsequent analysis.

### 2.5 Questionnaire Data and Statistical Analysis

Free‐text responses to the question “What do you understand by the term ‘ethnic group’?” were sorted according to whether they mentioned the following terms: ancestry, genetics, culture, place of origin, appearance, and race. Words such as skin tone, physical attributes, or color were categorized with appearance. Hereditary, genes, and genotype were categorized with genetics.

For the purposes of data presentation of the Likert‐scale responses in the pretest questionnaire, the summary response is given as agree if the number of agree plus strongly agree responses was greater than the sum of strongly disagree, disagree, and neutral. Similarly, the summary response for disagree was given if the sum of strongly disagree and disagree was greater than the sum of neutral, agree, and strongly agree. Otherwise, the summary was given as inconclusive.

To determine any change in student responses between the pre‐ and posttest responses, the Likert scale data in the pre‐ and postquestionnaire responses were converted to numerical form: strongly disagree = 1, disagree = 2, neutral = 3, agree = 4, and strongly agree = 5; and the pre‐ and postquestionnaire responses were matched using the identification number. Similarly, in the questions about drug treatment: only environmental = 1, mostly environmental = 2, both genetic and environmental = 3, mostly genetic = 4, and only genetic = 5. Analysis was performed with GraphPad Prism 9 using a Wilcoxon paired signed‐rank test to determine statistical significance (*p* < 0.05). A priori estimate of sample size (performed using GPower 3.1) using effect size of 0.3 (medium); *α* = 0.05 gave a required sample size of 64 for a statistical power of 0.8 and 111 for a statistical power of 0.95.

### 2.6 Focus Group Approach

Given ethnicity is more accurately a social construct, the focus group approach needed sufficient flexibility on the part of the research team to ensure data collection, and collection explored both areas of convergence and divergence in thinking on the term. We developed a semistructured prompt guide that specifically probed the way in which students made sense of the term ethnicity. The guide was refined following conversations within the research team to identify the specific components of race and ethnicity for investigation and pharmacokinetics examples where race and ethnicity were commonly used in teaching. Where relevant and necessary following completion of the questionnaire by students, student perceptions of relevant genetic mechanisms around pharmacokinetics were probed in‐depth to further illuminate the response recorded by them earlier in that part of the study. Focus group duration was 1 h.

### 2.7 The Focus Group Sample

All students in the first year of Graduate Entry Medicine and MSc Drug Discovery and second year of BSc programs were emailed an invitation to participate in the research. Students were informed that involvement was voluntary, and participation was not a formal requirement or mandatory for progression on the course program. Students interested in participating confirmed their agreement to attend a focus group with a member of the research team. Participants confirmed their agreement to attend and allow the researcher to capture the conversation using Microsoft Teams and disseminate the findings of the research in the form of presentations or manuscripts for peer‐review publication as appropriate. For logistical reasons, two focus groups were held in person and two were held via Microsoft Teams.

### 2.8 Focus Group Data Analysis

All interviews were recorded using MS Teams and the transcript was checked manually by listening back to the recording and correcting any transcription errors. A thematic analysis approach is used to make sense of participant perceptions [29]. Thematic analysis was chosen for the analytic approach due to the inherent flexibility afforded by the various techniques associated with it. The flexibility comes from being able to use either inductive or deductive strategies, enabling analysis to be informed by preexisting theories or frameworks [29].

The first step involved familiarization with the data following transcription. The data transcripts were read and re‐read in order to capture initial ideas and reactions to the conversation. The second step involved the generation of initial codes that identified features of interest within the data in a systematic fashion across the entire dataset. Thereafter, data relevant to each code were identified in a recursive manner. The third step involved searching for themes and specifically required the research team to collate codes into categories representing high‐level areas of interest. All data relevant to each theme were subsequently gathered together. The fourth step involved reviewing the themes within the research team and specifically checking the themes “worked” at multiple levels. The first level represented alignment between the coded extracts at the theme heading, and the second level represented alignment with the entire data set and the theme heading. The output of this fourth step was the generation of a thematic “map” of analysis. The fifth step required further scrutiny of the definition and naming of themes, so the specifics of each one were explicit, and contributed to the overall story of the analysis. Clear definitions and names for each theme resulted from this step before a final sixth step was undertaken. In this step, exemplar extract examples were selected that related back the analysis to the research question and the background literature for presentation in the Results section.

### 2.9 Ethics

The study was approved by the Faculty of Medicine and Health Sciences Research Ethics Committee of the University of Nottingham (Ethics Approval Number: FMHS 362‐1021). Respondents gave informed consent in accordance with the ethics approval.

## 3 Results

Participants (*n* = 112) from four courses in the Faculty of Medicine and Health Sciences (Year 1 Graduate Entry Medicine 56 students, Year 2 BSc Medical Physiology and Therapeutics 41 students, Year 2 BSc Pharmacology 5 students, and Drug Discovery MSc 10 students) completed the questionnaires.

### 3.1 Questionnaire Responses

Eighty‐one percent of participants had studied biology to age 18 (final year of secondary (high) school), 43% had previously studied biology, and 37% had previously studied genetics in an undergraduate University degree. The median test score was 83% (interquartile range 67%, 92%). Question‐level analysis revealed that students were least likely to correctly answer about the relationship between gene(s) and disease or gene(s) and traits as almost half of the students chose the incorrect answer for: “Human diseases caused by a single gene are more common than those caused by a combination of many genes.”

On the whole, students did not respond in a manner that was consistent with showing genetic deterministic beliefs (Table 2). In 4 of 6 statements (Table 2b–d,f), most students chose a response that did not indicate genetic deterministic beliefs. In the remaining two statements, there was either a bimodal distribution (Table 2a) or a large proportion of neutral responses (Table 2e). Where there was an inconclusive result, the educational intervention showed a significant effect with a shift toward a nongenetic deterministic viewpoint. After the educational intervention, for two statements where genes are related to traits and disease and the prequestionnaire responses were inconclusive, there was a statistically significant shift after the teaching away from the genetic deterministic view (Table 2, these statements were (a) “Cloning can produce a copy of an animal identical in all respects with the original—so you could recreate a much‐loved pet for example” and (e) “Genes have a greater role in most human disease than environmental factors do”).

**TABLE 2:** Summary of student responses to questionnaire statements about genes, traits, and disease and change after the educational intervention. A shift in the responses between the pre‐ and postquestionnaires was calculated by converting the responses to numerical values (from strongly disagree = 1 to strongly agree = 5) and the shift was calculated by subtracting a student's prequestionnaire response from their postquestionnaire response. Shifts were assessed using a Wilcoxon paired signed rank test performed with GraphPad Prism 9.

Statement	Prediction of genetic deterministic beliefs	Prequestionnaire responses	Shift in post‐compared with prequestionnaire responses
(a) Cloning can produce a copy of an animal identical in all respects to the original—so you could recreate a much‐loved pet for example	Agree	**Inconclusive** Strongly disagree 5% Disagree 37% Neutral 16% Agree 37% Strongly agree 5%	Significant shift (*p* = 0.0005) toward disagree
(b) Cloning could never produce a completely identical copy of a human being because our development is determined by much more than just our genes	Disagree	Strongly disagree 1% Disagree 3% Neutral 7% Agree 51% Strongly agree 38%	No change
(c) The children of musicians are more likely to become musicians themselves not because they inherit musical talent but because they follow their parents' example	Disagree	Strongly disagree 1% Disagree 12% Neutral 18% Agree 59% Strongly agree 11%	Significant shift (*p* < 0.0001) toward disagree
(d) Apart from changes that take place after birth and throughout their lifetime (such as accidental scars, hairstyle, clothing, and tattoos), it is not possible to tell identical twins physically apart	Agree	Strongly disagree 14% Disagree 49% Neutral 10% Agree 16% Strongly agree 11%	No change
(e) Genes have a greater role in most human diseases than environmental factors do	Agree	**Inconclusive** Strongly disagree 5% Disagree 30% Neutral 37% Agree 23% Strongly agree 4%	Significant shift (*p* < 0.0001) toward disagree
(f) Changes in lifestyle (diet, exercise, and so on) can never override a person's genetic risk factors	Agree	Strongly disagree 9% Disagree 47% Neutral 19% Agree 22% Strongly agree 4%	No change

**FIGURE 3:**
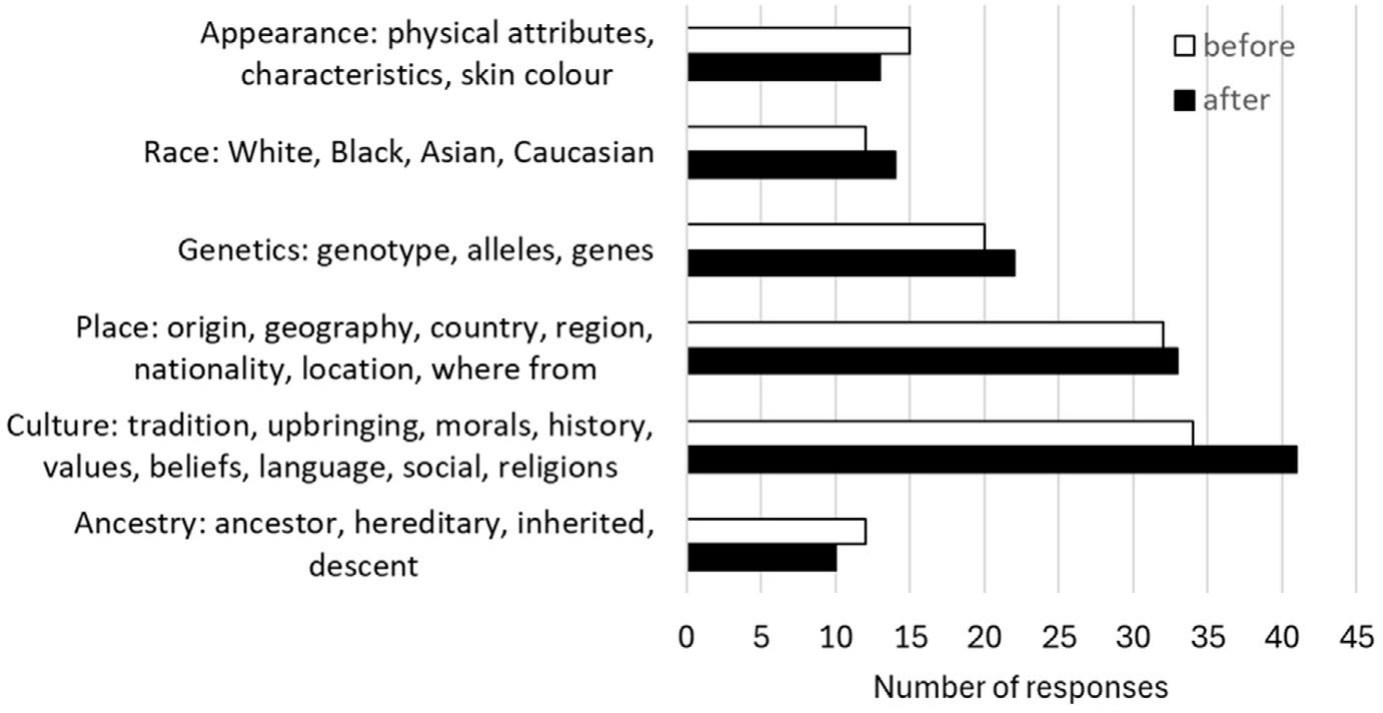
Students' definition of ethnicity in a free‐text response. Responses were categorized into six categories based on the free‐text responses. There were 87 individuals who answered this question in both the pre‐ and postquestionnaire. Some individuals used words from more than one category.

In response to a simple question asking for a free‐text response, students most commonly defined ethnicity in relation to culture and traditions followed by place of origin (Figure 3). Responses were grouped into six categories and students often mentioned several of these categories in their responses with a maximum of 5 categories mentioned, minimum of 1, and median of 2. The largest proportion of students (61% before and 67% after the teaching) defined ethnicity in terms of one or more cultures, traditions, places, appearance, or ancestry. Only 20 (23%) students mentioned genetics (or genes or genotype) when defining ethnicity prior to the teaching, and of these, half defined ethnicity as solely due to genetics with the remaining half of students using other words in addition, relating to culture, appearance, ancestry, or place of origin. Overall students' definition of the term ethnicity, as expressed in the questionnaire responses, did not change after the educational intervention.

When asked in the questionnaire to what extent they agreed with the statement “There is much more genetic variation within ethnic groups than between them,” the largest number (42%) of students disagreed with this statement prior to the educational intervention closely followed by the number who put neutral (40%) with only 18% agreeing. There was a statistically significant shift after the teaching (Wilcoxon signed rank, *p* < 0.0001) with more students agreeing (49%) than disagreeing (24%) or being neutral (27%).

**FIGURE 4:**
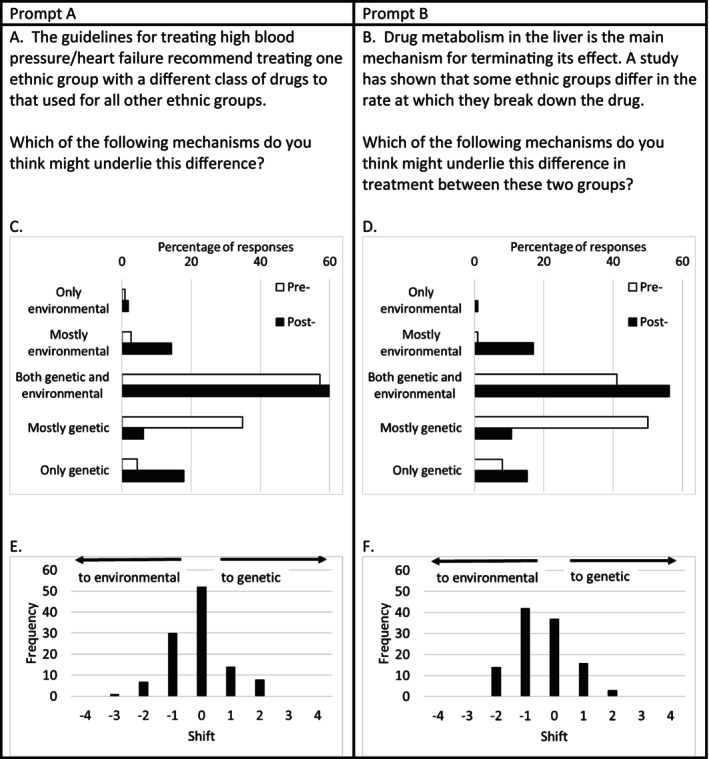
Summary of student responses to pre‐ and postquestionnaire statements about pharmacological mechanisms. Panels (A and B) show the prompts given in the questionnaires. Panels (C and D) show the percentage of responses (*n* = 112) for Prompts (A and B), respectively. Pretest data are in open columns; posttest data are in filled columns. Panels (E and F) show the shift between the pre‐ and postquestionnaire responses for Prompts (A and B), respectively. Responses were allocated numerical values, “only environmental” = 1, “mostly environmental” = 2, “both genetic and environmental” = 3, “mostly genetic” = 4, and “only genetic” = 5. The shift was calculated as the preresponse subtracted from the postresponse for each student. A positive shift reflects a move toward selecting a genetic mechanism and a negative shift reflects a move toward selecting an environmental mechanism. Difference between the pre‐ and postquestionnaire responses was assessed using a Wilcoxon paired signed rank test performed with GraphPad Prism 9. There was no significant shift in responses for Prompt (A). There was a significant shift (*p* < 0.0001) away from genetic and toward environmental for Prompt (B).

In questions regarding cardiovascular drug mechanisms, the largest proportion of students proposed both genetic and environmental mechanisms underlying ethnic differences in pharmacological treatment of hypertension (Figure 4) between Black and non‐Black patients, although there was a significant minority suggesting a genetic mechanism. The educational intervention had no effect on this response.

In contrast, when asked to propose a mechanism for differences in drug metabolism prior to the educational intervention, the majority of students proposed a genetic mechanism. After the educational intervention, there was a statistically significant shift away from a genetic mechanism toward environmental or both environmental and genetic (Figure 4), reflecting the key messages contained in the teaching.

### 3.2 Focus Groups

Four focus groups were held with 17 students in total (group sizes were 7, 2, 3, and 5 students) after the educational intervention about interindividual variation in pharmacokinetics. Focus group discussions showed that students' perception of ethnicity was heavily influenced by their previous experiences of going through life, and in particular, family background and upbringing; using examples that related to a group of people from a certain region (Table 3).

**TABLE 3:** Focus group themes and example quotes regarding how students understand ethnicity.

Idea	Quote
Relates to a group of people from a certain region	Whenever I have a form medical form to fill in, they never have [name of country]. You know, tick box it's always other Asian that I have to tick and which I don't really feel sort of surmises exactly where I'm from People wouldn't say they were Indian, they would say they were Gujarati or would say that they're Punjabi and that sort of thing and so that's like the first place that I think of when I think of a place where you know a lot of people might look at it and say Indian is an ethnicity, but then well, actually no, it's not.
Relates to the student's own family background and experiences	… it's history, it's your traditions … I'm from xxxx, we care a lot about our traditions, and when I think ethnicity, I don't think appearances as much as other people would, I think more of my cultural traditions. The reason that I focus more on genetics is because I think culture doesn't mean very much to me just because of how varied my background is. My grandparents are [nationality A] except one which is [nationality B] and ethnically we're xxxx family but religiously we're yyyy and then obviously I grew up in Britain.
Influenced by life experiences	I used to work in the ward that had people from lots of different sub regions of [name of country]. The more exposure you have, like different kinds of people, the more people you meet you have, like a stronger understanding of ethnicity. It's like going through primary school secondary school. Um, having all these friends from like different ethnicities and different backgrounds, I think just helps you understand what ethnicity means more and more.

When prompted to explain their answer to the question in the pre‐ and postquestionnaire about the extent to which they agreed with the statement “There is much more genetic variation within ethnic groups than between them,” students had both difficulty in defining ethnicity and difficulty in separating a regional context for ethnicity with the continental‐scale racial categorization of it (Table 4). Other students felt that they just did not know and had not been taught about it previously or, if they had, it had been quite simplistic (Table 3).

**TABLE 4:** Focus group themes and example quotes regarding genetic variation within or across different ethnic groups.

Using a regional and cultural context for ethnicity	I would say that there was more variance within. I'm just thinking back to different dialects within countries and continents. And people might sort of identify as different ethnicities based on their own dialect that they speak. And I would say for that reason there would be there would be more variation within, definitely. I am kind of neutral on it myself, largely because I can see that there's obviously a great variation between someone from Sub Saharan Africa and someone from East Asia. But then at the same time as that, how many different cultures are there in Sub Saharan Africa? There's lots hundreds and hundreds.
Difficulty with definitions	I left neutral because it depends on what group you're talking about and what you mean by genetic diversity. Could it be due to like their understanding of ethnicity, perhaps? It depends very much on what you mean by genetic variation and also what ethnic groups you're talking about.
Don't know	Genetic differences between people aren't taught so much on the course, or at least we haven't been taught about it so much just yet. I don't think there was much talk about how certain genes and things affect certain ethnicities. They'll be like, you know, mention of this will affect this certain group more. Here's the data, but nothing more than that.
Previous teaching focused on simple genetics when teaching about disease.	We're taught that [genetics] more, and I think that's why maybe we have a bias towards the genetics. I think they taught us the kind of genetic inheritance through blood types, mostly blood type. So yes, yeah we had. Yeah yeah. I think sickle cell, but I can't remember.
Recognized a misconception after attending the teaching intervention or during previous degree	I remember when we did that session, the initial questionnaire session. I was so upset that … that I've gone my whole life thinking that like that, like black people genetics was different enough that you would want to treat them differently. I always thought everything is mainly down to genetics because it's very difficult for environmental factors to change the very hardware that your body basically yeah is running on, so it's like that was always my opinion, but now I definitely see that there's so many environmental factors that can influence it in a sense, not change it, but definitely influence it. Before starting my previous degree, I thought was mostly genetics. You know, certain people get type one diabetes ‘cause they have the genes. That's all I thought. But then during it we did a lot about environment in genetics and different aspects of it.

## 4 Discussion

The majority of our students understand ethnicity as an individual's culture, traditions, and place of origin with some including ancestry, genetics, and related terms. We also found that the term ethnicity could be used with different meanings, ranging from the personal/family level right through to the large‐scale continental groups, sometimes expressed as race, depending on the context and the prompts. We found little evidence of genetic deterministic views but considerable confusion about the relationship among genes, environment, and disease. While the educational intervention was able to influence students' views about ethnicity in relation to pharmacokinetics, it did not influence students' overall understanding of ethnicity or their tendency to ascribe genetic mechanisms to observations about ethnic differences in choice of drug in cardiovascular pharmacology.

Students defined ethnicity largely in terms of culture (e.g., traditions and beliefs) and place (e.g., where from and place of origin) and this is consistent with other definitions such as that of the United Nations [13], the National Academies of Sciences, Engineering, and Medicine [12], and major dictionaries [30]. This is in contrast to the use of the term ethnicity in the pharmacological literature where ethnicity is used to describe racial groups, that is, Caucasian, African American, and Asian. Indeed, in a recent scoping review [5], 50 of 62 papers on ethnic differences in pharmacokinetics refer to ethnicities as racial groups, that is, Caucasian, African American, Asian, White, and Black. This observation highlights the potential for confusion or cognitive dissonance when students understand ethnicity in a different way to the one in which it is used in teaching pharmacology. This suggests a need for clarity in how the terms ethnicity and race are used and recognition that students may bring a range of ideas to the classroom. Gaining clarity in this area will likely be challenging as the terms race and ethnicity are used in a range of ways which can vary with context and there is considerable overlap in the meanings of the two terms [31, 32]. Nevertheless, teaching about the historical construction of racial categories [33] has been shown to influence the way students understand race and ethnicity [34], and devoting some time to this is an important area of future improvement in our curriculum.

The life experiences and the ethnic composition of the student cohort may also play a role in refining how students understand ethnicity. Our students are drawn from an ethnically diverse cohort and some students commented that this affected the way they viewed ethnicity. Some of the students had significant life experiences gained through traveling, and this gave them ideas about ethnicity outside of their immediate family. This demonstrates the value of diverse life experiences among the student cohort and raises the proposition that students can learn about ethnicity from their peers using facilitated or structured discussions. Creating supportive environments for open and nonjudgemental conversations has been proposed in a student–staff cocreation project as a way of improving this area of the curriculum [35]. Furthermore, the inclusion of cultural humility into the curriculum encourages an openness to learn from others [36].

The questionnaires used in this study were designed to ascertain students' prior knowledge and understanding of genetics and genetic determinism. In contrast to previous studies, a number of which used a questionnaire approach, we were unable to detect significant genetic determinism in our cohort [14, 15, 28, 37, 38]. Our study is different from the previous studies in a number of ways, not least that we are using a different questionnaire, students are in a pharmacology classroom rather than genetics, and we are situated in the United Kingdom rather than the United States. Since some of our participants have a previous degree in Biological Sciences, it is possible that our cohort, on the whole, has a relatively high genomics literacy, and Donovan et al. [15] have shown that students with greater knowledge of genomics are less likely to show genetic deterministic views.

Both questionnaire‐ and focus‐group approaches have their limitations. Genetic determinism has typically been measured with a questionnaire designed for use in genetics teaching which encompassed aspects of genetics and genomics technologies all of which were outside the scope of our subject teaching [37, 38, 39, 40, 41]. There does not appear to be a validated questionnaire that has been extensively used by several groups. It was for this reason that we explored the questionnaire findings through the use of focus groups. Similarly, a focus group approach has limitations in that views are obtained from a smaller number of students. The students attending the focus groups were self‐selecting and it is likely that those who attended were more motivated and interested in discussing issues relating to ethnicity. Given that we saw that students' life experiences influenced their ideas about ethnicity, it is likely that the ethnic composition of our cohort will influence our results. We also know that there are differences in the way ethnicity and race are conceptualized in different countries [13, 32, 40]. This points to the need to perform a larger multi‐institutional study to address this question and the generalizability of our findings.

The observation that students were most likely to choose a genetic mechanism for ethnic differences in drug metabolism could indicate the presence of a misconception that there may be greater genetic similarity within an ethnic group than between one ethnic group and another, that is, ethnic groups are genetically distinct [15]. Genetic similarity is a “quantitative measure of the genetic resemblance between individuals that reflects the extend of shared genetic ancestry” [12, 42]. The total number of gene variants in the human genome found within ethnic groups, that is, African, Asian, and European, has an extensive degree of overlap reflecting the findings that these groups share many of the same within‐group gene differences. Another way of looking at this is that genetic variation among humans is continuous and does not map to racial categories [15, 43].

Human geneticists have called for use of ethnic group terms such as White British and Black African in the context of biological variation to stop since these terms, which were introduced centuries ago, are not relevant for understanding population genetics [44, 45]. Likewise, Bhopal and Donaldson [46] argue that use of these racial terms encourages “division of society by skin color, reinforcing racial stereotyping, and hides a remarkable heterogeneity of cultures,” an observation which was echoed by students during focus groups. Following this line of argument, it is logical to suggest that pharmacology educators should avoid the use of these terms in the pharmacology classroom in the context of biological variation.

There are several potential reasons for students' confusion about genetic variation within and between ethnic groups across both questionnaire and focus group formats. First is that genetic essentialism, the belief that race is an innate attribute that defines the characteristics of its members, is a common and spontaneous way of thinking [32, 39]. Second, it is possible that our students have been influenced by general discourse across society, perhaps more so now by personalized genetics companies advertising ancestry DNA tests that play on the idea that one's race or ethnicity can be discovered through genetic ancestry testing, thereby placing a biological basis to race and ethnicity [47]. Third, students may be uncertain as to whether we mean family‐level ethnicity or continental‐scale racial groups. While these are possibilities, our students pointed out that much of their previous teaching about disease mechanisms had invoked genetic mechanisms and this foregrounding of genetics influenced their responses, that is, “genetics by default.” This adds further weight to the calls for significant changes to the way genetics is taught at schools and universities to reduce the potential for inadvertently giving ethnicity and race a biological basis [28, 48, 49]. For example, teaching about sickle cell disease in the context of its prevalence in areas with high levels of malaria rather than simply as a disease of Black people [50]. It also underlines the importance of including in our educational intervention the points that individuals vary due to a variety of factors both genetic and nongenetic, that allele prevalence is not exclusive to a particular ethnic group, and therefore ethnicity should not be used as a proxy for genetics.

Our introduction of an educational intervention about interindividual variation allowed us to tackle potential misconceptions about ethnicity and genetics head‐on and offer a range of alternative mechanisms for interindividual variation in pharmacokinetic processes. Where students did have genetic deterministic views, these were overall reduced by the educational intervention possibly because it highlighted nongenetic mechanisms for variation between individuals. Some, but not all, students clearly changed their prior conceptions about ethnic differences in relation to pharmacokinetics with a greater appreciation of the range of ways individuals can vary. However, the fact that these students did not transfer this understanding to another aspect of pharmacology, drug treatment for hypertension, suggests that it is important to address issues relating to ethnicity wherever they arise and to ensure that ethnicity or race are not presented as biological categories. The fact that not all students moved away from a genetic essentialist view of ethnicity and race suggests that it is important to reinforce these ideas across the whole medical or medical sciences curriculum. This is supported by a number of authors who have underlined the importance of being clear about how ethnicity and race are portrayed in preclinical medical education [51, 52].

## 5 Conclusion

We have designed and shared a new teaching resource about interindividual variation in pharmacokinetics which has allowed us to directly address misconceptions about ethnic differences and appreciate that a range of factors, both genetic and nongenetic, can influence interindividual variation in response to medication. Through investigating what our students bring as prior knowledge and understanding, we have realized the degree of confusion regarding the meaning of ethnicity and race and confusion around the degree of genetic similarity between racial or ethnic groups. This has highlighted the importance of clearly defining ethnicity or race in a given context and teaching explicitly that ethnicity and race are socially constructed and self‐determined and that they are not defined by genetic similarity at the continental scale. It has become increasingly clear that we need to re‐evaluate race and ethnicity across the whole curriculum and take a holistic approach encompassing the historical development of these concepts and their impact on social and structural determinants of health.

## Author Contributions

All authors contributed to the study conception and design. O.O. and J.A.K. prepared and delivered the educational interventions and questionnaires, and the focus groups were led by R.P. Data collection and analysis were performed by J.A.K. and O.O. The first draft of the manuscript was written by J.A.K. and all authors commented on previous versions of the manuscript. All authors read and approved the final manuscript.

## Ethics Statement

The questionnaire and methodology for this study were approved by the Faculty of Medicine and Health Sciences Research Ethics Committee of the University of Nottingham (Ethics Approval Number: FMHS 362‐1021).

## Consent

Informed consent was obtained from all individual participants included in the study.

## Conflicts of Interest

The authors declare no conflicts of interest.

## Supporting information


**Supporting Information S1.** Inter‐individual variability in PK teaching resource.

**Supporting Information S2.** Questionnaire.

## Data Availability

The data sets generated during and/or analyzed during the current study are available from the corresponding author upon reasonable request.
