# Supplementary material for: Addressing Ethnicity in the Design and Evaluation of an Educational Intervention on Interindividual Variation in Pharmacokinetics
Source: Pharmacol Res Perspect. 2025 Feb 6;13(1):e70073. doi: 10.1002/prp2.70073 (PMC11800234; doi:10.1002/prp2.70073)
Supplement: Supplementary file 2 — Supporting Information S2. Questionnaire. [file PRP2-13-e70073-s002.docx]

**S2: Supplementary Material to Koenig, Olafuyi and Patel, Addressing ethnicity in the design and evaluation of an educational intervention on inter-individual variation in pharmacokinetics.**

**Pre-Questionnaire**

**Section 1 : Background Information**

| 1. Please enter the last five digits of your student ID number. This is so that we can match up your responses with those from a second questionnaire later. Once we have matched up the responses we will delete these digits from our records so that the data will be stored with anonymous responses. |  | |
| --- | --- | --- |
| The following questions ask about your educational background. Please select yes or no. | Yes | No |
| 1. Did you study biology up to age 16? (e.g. a GCSE in biology or combined science) |  |  |
| 1. Did you study biology up to age 18? (e.g. A level, IB or Scottish Highers) |  |  |
| 1. Have you studied biology at university undergraduate level prior to your current degree course? |  |  |
| If yes, have you studied genetics? |  |  |
| 1. Have you studied biology at postgraduate level prior to your current degree course? |  |  |
| If yes, have you studied genetics at postgraduate level? |  |  |

**Section 2: Your opinions**

| Cloning can produce a copy of an animal identical in all respects with the original— so you could recreate a much-loved pet for example. | Strongly agree | agree | Neutral | disagree | Strongly disagree |
| --- | --- | --- | --- | --- | --- |
| The children of musicians are more likely to become musicians themselves not because they inherit musical talent but because they follow their parents’ example | Strongly agree | agree | Neutral | disagree | Strongly disagree |
| Apart from changes that take place after birth and throughout their lifetime (such as accidental scars, hair style, clothing, tattoos, etc.) it is not possible to tell identical twins physically apart | Strongly agree | agree | Neutral | disagree | Strongly disagree |
| Genes have a greater role in most human disease than environmental factors do | Strongly agree | agree | Neutral | disagree | Strongly disagree |
| Changes in lifestyle (diet, exercise and so on) can never override a person’s genetic risk factors | Strongly agree | agree | Neutral | disagree | Strongly disagree |
| Cloning could never produce a completely identical copy of a human being because our development is determined by much more than just our genes | Strongly agree | agree | Neutral | disagree | Strongly disagree |
| What do you understand by the term “ethnic group”? | Free text response | | | | |
| The guidelines for treating high blood pressure recommend treating one ethnic group with a different class of drugs to that used for all other ethnic groups. Which of the following mechanisms do you think might underly this difference? | Only environ-mental | Mostly environ-mental | Both environ-mental and genetic | Mostly genetic | Only genetic |
| Midazolam is a drug used in the treatment of anxiety. It is broken down in the liver and this is the main mechanism for terminating its effect. A study has shown that some ethnic groups differ in the rate at which they break down midazolam. Which of the following mechanisms do you think might underly this difference in treatment between these two groups? | Only environ-mental | Mostly environ-mental | Both environ-mental and genetic | Mostly genetic | Only genetic |

**Section 3: Your knowledge**

The following section asks about your general knowledge in this area. You may or may not have studied this in your course so far so answer as best you can from your general knowledge.

|  | **True** | **False** |
| --- | --- | --- |
| Cells of different types (e.g. nerve cells, liver cells) in the same person contain different genetic information |  |  |
| The genetic information is organized into separate units (genes) which influence different aspects of our development |  |  |
| The environment has no effect on the way in which our genes influence our characteristics |  |  |
| Many genes code for proteins which have important physiological functions in the body, such as haemoglobin in the blood, or insulin, which controls blood sugar levels |  |  |
| If a woman is identified as having a BRCA1 mutation, she will develop breast cancer at some point in her life |  |  |
| Two parents are carriers for the recessive disorder, cystic fibrosis, therefore there is a 1 in 4 chance that a child they have will have the disease. If the couple already have 3 unaffected children, a 4th child would be more at risk than the 1st child |  |  |
| There is a separate gene for each characteristic of a human being |  |  |
| Overall, more human disease is caused by environmental factors (such as diet, lifestyle, exposure to bacteria, viruses or chemicals) than by genetic factors |  |  |
| Human diseases caused by a single gene are more common than those caused by a combination of many genes |  |  |
| Cells of the same type (e.g. brain cells) in different people contain the same genetic information |  |  |
| Mutations in the DNA always lead to diseases/disorders |  |  |
| The variety we see in humans can be completely explained by genetic differences |  |  |
